# miR-223 accelerates lipid droplets clearance in microglia following spinal cord injury by upregulating ABCA1

**DOI:** 10.1186/s12967-024-05480-5

**Published:** 2024-07-15

**Authors:** Zhilin Ou, Yongquan Cheng, Hao Ma, Kai Chen, Qiong Lin, Jiayu Chen, Ruqin Guo, Zhiping Huang, Qixian Cheng, Nima Alaeiilkhchi, Qingan Zhu, Zucheng Huang, Hui Jiang

**Affiliations:** 1 Division of Spine Surgery, Department of Orthopaedics, Nanfang Hospital, Southern Medical University, Guangzhou, 510515 Guangdong China; 2 The First School of Clinical Medicine, Nanfang Hospital, Southern Medical University, Guangzhou, 510515 Guangdong China; 3 School of Anesthesiology, Southern Medical University, Guangzhou, 510515 Guangdong China; 4 International Collaboration on Repair Discoveries (ICORD), Blusson Spinal Cord Centre, University of British Columbia, Vancouver, Canada

**Keywords:** Spinal cord injury (SCI), Microglia, Lipid droplets, miR-223, ATP-binding cassette transporter A1 (ABCA1)

## Abstract

**Background:**

Spinal cord injury (SCI) is characterized by extensive demyelination and inflammatory responses. Facilitating the clearance of lipid droplets (LDs) within microglia contributes to creating a microenvironment that favors neural recovery and provides essential materials for subsequent remyelination. Therefore, investigating MicroRNAs (miRNAs) that regulate lipid homeostasis after SCI and elucidating their potential mechanisms in promoting LDs clearance in microglia have become focal points of SCI research.

**Methods:**

We established a subacute C5 hemicontusion SCI model in mice and performed transcriptomic sequencing on the injury epicenter to identify differentially expressed genes and associated pathways. Confocal imaging was employed to observe LDs accumulation. Multi-omics analyses were conducted to identify differentially expressed mRNA and miRNA post-SCI. Pathway enrichment analysis and protein-protein interaction network construction were performed using bioinformatics methods, revealing miR-223-*Abca1* as a crucial miRNA-mRNA pair in lipid metabolism regulation. BV2 microglia cell lines overexpressing miR-223 were engineered, and immunofluorescence staining, western blot, and other techniques were employed to assess LDs accumulation, relevant targets, and inflammatory factor expression, confirming its role in regulating lipid homeostasis in microglia.

**Results:**

Histopathological results of our hemicontusion SCI model confirmed LDs aggregation at the injury epicenter, predominantly within microglia. Our transcriptomic analysis during the subacute phase of SCI in mice implicated ATP-binding cassette transporter A1 (*Abca1*) as a pivotal gene in lipid homeostasis, cholesterol efflux and microglial activation. Integrative mRNA-miRNA multi-omics analysis highlighted the crucial role of miR-223 in the neuroinflammation process following SCI, potentially through the regulation of lipid metabolism via *Abca1*. In vitro experiments using BV2 cells overexpressing miR-223 demonstrated that elevated levels of miR-223 enhance ABCA1 expression in myelin debris and LPS-induced BV2 cells. This promotes myelin debris degradation and LDs clearance, and induces a shift toward an anti-inflammatory M2 phenotype.

**Conclusions:**

In summary, our study unveils the critical regulatory role of miR-223 in lipid homeostasis following SCI. The mechanism by which this occurs involves the upregulation of ABCA1 expression, which facilitates LDs clearance and myelin debris degradation, consequently alleviating the lipid burden, and inhibiting inflammatory polarization of microglia. These findings suggest that strategies to enhance miR-223 expression and target ABCA1, thereby augmenting LDs clearance, may emerge as appealing new clinical targets for SCI treatment.

**Supplementary Information:**

The online version contains supplementary material available at 10.1186/s12967-024-05480-5.

## Background

SCI constitutes a devastating insult to the central nervous system (CNS), potentially resulting in compromised motor, sensory, and autonomic neural functions. Following SCI, the accumulation of myelin debris at the injury epicenter is accompanied by the activation of resident microglia and the infiltration of blood-derived macrophages [1, 2]. These cells are inherently heterogeneous and display remarkable functional plasticity, categorizing into two major phenotypes: M1 and M2 [3, 4]. They are capable of transitioning under the influence of surrounding microenvironment [5]. M1 macrophages/microglia induce tissue inflammation and damage by releasing pro-inflammatory cytokines, reactive oxygen species (ROS), and nitric oxide (NO). Conversely, M2 cells secrete anti-inflammatory factors and exhibit reduced capacity in pro-inflammatory molecule production, thus promoting wound healing and tissue remodeling. After SCI, the preponderance of M1 over M2 macrophages leads to chronic inflammation and secondary damage [6, 7]. Although macrophages are potent phagocytes, their efficiency in processing ingested material is suboptimal [8]. Therefore, microglia play a more crucial role as “professional phagocytes” followed by SCI. Long-term demyelination occurs post-SCI, with lipid-containing myelin debris being absorbed and processed by inflammatory cells, including microglia—a process crucial for paving the way for regenerative reactions and inflammation resolution [9–11]. The high cholesterol content in myelin debris exceeds the phagocytes’ efflux capabilities, causing an accumulation of LDs and cholesterol crystal-laden foam-like microglia within the lesion site [12]. Scholars have termed microglia with substantial LDs accumulation as “lipid-droplet-accumulating microglia” (LDAM) [13]. These cells exhibit defective phagocytic functions, generate abundant ROS, and secrete pro-inflammatory cytokines. Moreover, they activate complement-mediated inflammatory pathways, which enhance the activation of M1-type cells. Consequently, the regulation of lipid homeostasis in microglia after SCI holds significant promise for therapeutic interventions in neuroprotection, tissue recovery, and neural prognosis.

The clearance of myelin debris and subsequent remyelination are crucial for neural regeneration following SCI [14]. Accumulated myelin debris has been demonstrated to impede white matter repair by directly inhibiting remyelination or promoting secondary inflammatory cascades [15]. Myelin is lipid-rich, with a significant cholesterol content that is resistant to degradation, thus necessitating storage and efflux mechanisms [16–19]. Recent studies have proposed that “recycling” cholesterol from myelin debris, through redistribution via lipoprotein particles, could meet the cholesterol requirements for remyelination by oligodendrocytes [20]. Therefore, restoring the ability of microglia to degrade and process cholesterol and LDs derived from myelin debris is considered as a promising therapeutic strategy to promote remyelination. ABCA1 is instrumental in this process, as it mediates the efflux of cholesterol and phospholipids to apolipoprotein A-I (ApoA-I) and apolipoprotein E (ApoE), culminating in the formation of nascent high-density lipoprotein (HDL) [21, 22]. This mechanism is pivotal in controlling intracellular lipid content and serves as a primary “gatekeeper” for reverse cholesterol transport in cells [23]. Hence, upregulating ABCA1 expression on microglial cell membranes may enhance the degradation of myelin debris and the clearance of LDs, induce a polarization towards the M2 anti-inflammatory phenotype, and provide raw materials for remyelination by oligodendrocytes. This presents a promising avenue for the treatment of SCI.

miRNAs are a highly conserved class of small RNA sequences, ranging in length from 20 to 23 nucleotides. They regulate gene expression by binding to the 3’-untranslated region (UTR) of target messenger RNAs (mRNAs) [24]. In the context of SCI, miRNAs have been identified as critical regulators and fine-tuners of various signaling pathways in SCI, playing roles in secondary injury and repair processes post-SCI [25]. Dysregulated miRNAs can participate in inflammation, inhibition of cell apoptosis, and modulation of axon regeneration through multiple pathways after SCI. For instance, miR-22-3p has been shown to promote intrinsic neuronal regeneration, restoring sensory conduction in the injured spinal cord [26]. Additionally, it enhances the polarization of M2 macrophages by suppressing interferon regulatory factor 5 (IRF5), thereby reducing spinal cord ischemia-reperfusion injury (SCIRI) [27]. miR-124, a neural-specific miRNA, holds potential for preventing and treating persistent inflammation and neuropathic pain post-SCI [28]. miR-146a inhibits inflammation and promotes SCI repair by the TLR/NF-κB signaling pathway [29]. miR-21 reduces cell apoptosis and inflammation after SCI, protecting neurons and facilitating post-injury recovery [30, 31]. Furthermore, miR-223 suppresses receptor-interacting protein 3 (RIP3)-mediated necrosis and the secretion of inflammatory factors, ultimately alleviating SCI [32]. Despite these known roles of miRNAs in SCI, the regulatory role of miRNAs in lipid homeostasis in microglia during SCI is underexplored. Therefore, our investigation aims to elucidate the regulatory mechanisms by which miRNAs influence lipid homeostasis in the context of SCI, thus contributing to a deeper understanding of the pathological mechanisms underlying this condition. In addtion, it may provide new insights for promoting the clearance of LDs in microglia post-SCI and creating a microenvironment conducive to neural repair.

In this study, we investigated the roles and functions of differentially expressed miRNAs (DEMs) and differentially expressed genes (DEGs) in SCI through bioinformatics analysis. Considering the interplay between lipid metabolism and inflammatory responses in the pathogenesis of SCI, we particularly explored the impact of miR-223, especially its functions in promoting LDs clearance and suppressing inflammation in microglia. We utilized a BV2 microglial cell line overexpressing miR-223 to assess myelin debris and LDs accumulation, expression of related targets, and inflammatory factors, with the aim of elucidating miR-223’s regulatory role on microglial lipid homeostasis.

## Materials and methods

### Animals

All experimental procedures were conducted in accordance with the ethical guidelines and approved by the Laboratory Animal Care and Use Committee of Nanfang Hospital, Southern Medical University. Adult male C57BL/6 mice aged 6–8 weeks (Experimental Animal Center of Southern Medical University, Guangzhou, China) were used in this study to ensure consistency and reduce variability in physiological responses due to hormonal fluctuations and age differences. They were housed in groups of five per cage under standard conditions, with free access to food and water and a 12:12 h light/dark cycle.

### C5 hemicontusion injury models establishment

The mice were anesthetized using isoflurane (3% for induction and 1.5–2% for maintenance), and C5 hemicontusion injuries were induced following a previously established protocol [33]. In brief, the C5 lamina was exposed and removed. Subsequently, a contusion SCI was generated at C5 using an impactor tip (diameter = 1.0 mm) with a preset displacement of 1.2 mm at 300 mm/s. This process was carefully controlled using an electromagnetic servo material testing machine (Instron E1000, Instron, United States). Biomechanical parameters, including actual impact displacement depth, contusion velocity, and maximum load force, were collected using sensors installed on the testing apparatus. The results indicate that the SCI group had an average impact displacement depth of 1.203 ± 0.021 mm, an average contusion velocity of 304.079 ± 3.853 mm/s, and an average maximum load force of 0.728 ± 0.067 N. For the sham surgery, only C5 laminectomy was performed. Post-injury, mice received appropriate care, including analgesics and hydration, to minimize discomfort and promote recovery.

### Tissue preparation

Two weeks after the injury, the mice were deeply anesthetized with pentobarbital sodium (80 mg/kg), perfused with 20 mL of 0.01 M phosphate-buffered saline (PBS) and fixed with 20 mL of 4% paraformaldehyde solution. Following perfusion fixation, the spinal cords were excised, and the excision scope encompassed both the injured segments and adjacent head-to-tail segments approximately 1 cm in length along the spinal cord. The specimens were immersed overnight in a 4% paraformaldehyde, dehydrated using an increasing sucrose gradient (12%, 18%, and 24%), embedded in OCT and frozen, and sectioned using a cryostat microtome.

For animals designated for histological analysis, fresh spinal cord tissue centered around the injury focus was promptly collected after PBS perfusion. This tissue was either immediately sequenced or stored in the freezer at -80 ℃.

### Immunofluorescence (IF) staining

The specimens were embedded in optimal cutting temperature compound (TissueTek, 4583, Sakura) and transversely sectioned into 20 μm slices using a Leica CM1950 cryostat. Cryofixed spinal cord sections were permeabilized and blocked in PBST with 1% BSA for 1 h at room temperature. The slices then were incubated overnight at room temperature with primary antibodies including anti-Iba1 (1:300, Abcam ab178846), anti-IL-1β (1:300, Abcam ab156791), anti-TMEM119 (1:300, Proteintech 27585-1-AP) and anti-IL-6 (1:300, Proteintech 66146-1-Ig).The following day, the slices was washed three times in PBS and incubated at room temperature for two hours with secondary antibodies: goat anti-mouse Alexa Fluor 488 (1:200, Abcam ab150113) and goat anti-rabbit Alexa Fluor 647 (1:200, Abcam ab150079). For lipid droplet staining, the slices were incubated with BODIPY (1:400, Invitrogen D3835) for 20 min post-primary antibody incubation. Subsequently, they were washed three times with PBS and mounted with Fluoromount-G (0100 − 20, Southern Biotech). To determine the lesion epicenter, we used serial spinal cord sections to identify the area with the greatest extent of tissue destruction and the largest damage area. This region is typically located at the midpoint of the injury range. Imaging was acquired and analyzed using a Zeiss confocal microscope (LSM980, Zeiss, Germany) and ZEISS ZEN 3.3 software.

BV2 cells were cultured on glass coverslips in 24-well plates. Following various treatments, the cells were fixed with 4% paraformaldehyde at room temperature for 20 min, and washed with PBST three times. After permeabilization with 0.1% TritonX-100/PBS for 15 min, the cells were rinsed with PBS, sealed in 5% BSA/PBS for 1 h at room temperature, and then incubated overnight at 4 °C with primary antibodies for anti-ABCA1 (1:300, Abcam ab307534), anti-Myelin Basic Protein (MBP, 1:300, Abcam ab7349), anti-IL-6 (1:300, Proteintech 66146-1-Ig), anti-iNOS (1:300, Abcam ab210823), anti-Arg1 (1:300, Proteintech 66129-1-Ig) and anti-TNFα (1:300, Abcam ab1793). Following PBS washes, cells were incubated with secondary antibodies including goat anti-mouse Alexa Fluor 488 (1:200, Abcam ab150113), goat anti-rabbit Alexa Fluor 555 (1:200, Abcam ab150078), goat anti-mouse Alexa Fluor 555 (1:200, Abcam ab150114) and goat anti-rabbit Alexa Fluor 647 (1:200, Abcam ab150079) for two hours. For LDs staining, sections were incubated with BODIPY for 20 min after secondary antibody incubation, followed by three PBS washes and nuclear staining with DAPI-containing fluorine-shielded sections. Imaging and analysis were conducted using a Zeiss confocal microscope (LSM980, Zeiss, Germany), Zeiss fluorescence microscope (Axio Imager D2, Zeiss, Germany) and Zeiss ZEN 3.3 software.

### Bioinformatics analysis

To extract total RNA, the spinal cord was dissected at the center of the lesion 14 days post-SCI. Whole transcriptome sequencing was employed to screen for differentially expressed DEGs and DEMs in the spinal cord tissue. The t-test was applied to filter differentially expressed genes, selecting with DEMs and DEGs based on P values < 0.01, log fold change (FC) ≥ 2, and log FC > average (log FC) + 2 × SD (log FC) [34]. Volcano maps and heat maps illustrating the differential expression of DEGs and DEMs across different samples were generated using the plot and heatmap packages in R Studio. The miRTarBase (http://mirtarbase.mbc.nctu.edu.tw/) and miRWalk (http://mirwalk.umm.uni-heidelberg.de/) databases were utilized to predict the targeted mRNAs of the identified DEMs. Subsequently, the predicted mRNAs of DEMs were further filtered by matching with the previously selected DEGs, resulting in the identification of DEM-DEG pairs. A miRNA-mRNA network was constructed by compiling all the selected DEM-DEG pairs, and Cytoscape software (version 3.10.0) was employed for visualization. Node degrees of the regulatory network were calculated simultaneously. Moreover, Gene Ontology (GO) and Kyoto Encyclopedia of Genes and Genomes (KEGG) analyses were performed using the enrichment analysis tools DAVID (https://david.ncifcrf.gov/), Metascape (https://metascape.org/), miEAA 2.1 (https://ccb-compute2.cs.uni-saarland.de/mieaa/) and DIANA miRPath v4.0 (https://diana-lab.e-ce.uth.gr/app/miRPathv4/). A P value < 0.01 was considered statistically significant.

### Myelin debris isolation

Myelin debris was purified from post-mortem mouse brain tissue using density gradient centrifugation as previously described [35]. Myelin protein concentration was determined using the BCA Protein Assay Kit (Thermo Fisher), according to the manufacturer’s guidelines. The endotoxin content in the isolated myelin was confirmed to be negligible using a Chromogenic Limulus Amebocyte Lysate (LAL) assay kit (Genscript Incorporation).

### Cell culture and treatment

BV2 cells were employed extensively to induce cell injuries, emulating an in vitro model of SCI [36]. The BV2 cell line was procured from the Shanghai Cell Research Center (Shanghai, China) and cultured in DMEM/F12 medium (Gibco; Thermo Fisher Scientific, Inc., Waltham, MA, USA) supplemented with 10% FBS (Gibco) and 1% penicillin-streptomycin (Sigma-Aldrich, St. Louis, MO, USA) in a 5% CO2 atmosphere at 37 °C. Upon reaching approximately 80% confluence, cells were detached using trypsin and passaged for subsequent experiments. BV2 cells were then stimulated with 100 µg/ml of myelin debris and 100 ng/ml of lipopolysaccharide (LPS) (Sigma-Aldrich, St. Louis, MO, USA) at 37 °C for 24 h.

### Cell transfection

BV2 cells were seeded in a 6-well plate at a density of 1 × 10^5 cells per well. Upon reaching 80% confluence, the culture medium was replaced with serum-free medium, and the cells were cultured for an additional 24 h. Subsequently, the cells were infected with lentiviral vectors at an appropriate multiplicity of infection (MOI). The lentiviral vector (Shanghai Hanyin) was used to construct PSLenti-EF1-mCherry-P2A-Puro-CMV-Mir223-WPRE (miR-223 OE), which resulted in an increase of approximately 1.2 to 1.6 times compared to the negative control (NC) group. Additionally, a control construct containing the empty lentiviral vectors for miR-223 (miR-223 NC) was generated.

### Western blot analysis

Western blot analysis was conducted following established protocols. In brief, 40 µg of protein samples extracted from BV2 cells were transferred onto a PVDF membrane (Millipore). Subsequently, the membrane was blocked with 5% skim milk for 2 h at room temperature, followed by incubation with primary antibodies against ABCA1 (Abcam ab307534), iNOS (Abcam ab210823), Arg1 (Proteintech 66129-1-Ig), IL-1β (Abcam ab156791), TNF-α (Abcam ab1793) and β-tubulin (Proteintech 66240-1-Ig) at 4 °C overnight, with a dilution ratio of 1:1000. After primary antibody incubation, the blots were exposed to the corresponding secondary antibodies (Proteintech SA00001-1/2, 1:1000) for 1 h at room temperature. Protein bands were visualized using an ECL kit (GE Healthcare), and quantification of blot bands was performed using ImageJ version 1.46 (Rawak Software, Inc., Munich, Germany).

### Analysis of cholesterol efflux

BV2 cells were cultured in 6-well plates until they reached approximately 80% confluence. The medium was then replaced with serum-free medium, and the cells were incubated for 12 h and 24 h, respectively. The supernatant was collected. The cells were washed twice with PBS to remove the medium serum, then resuspended in 0.1 mL of lysis buffer per 1 × 10^6 cells and incubated at room temperature for 10 min. The lysates were heated at 70 °C for 10 min, then centrifuged at 2000 g for 5 min at room temperature. Total cholesterol content was measured using a cholesterol assay kit (E1015; Applygen Technologies Inc). The working solution was prepared by mixing reagent R1 with reagent R2 in a 4:1 ratio. A 5 mM cholesterol standard was diluted with anhydrous ethanol to concentrations of 2500, 1250, 625, 312.5, 156, 78, and 39 µmol/L. The microplate wells were quickly sealed, and 190 µL of the working solution was added to each well, followed by 10 µL of either the blank control (anhydrous ethanol), standard, or sample. The plate was incubated at 37 °C for 20 min and the optical density (OD) at 550 nm was measured. Cholesterol content was corrected by protein concentration per milligram, and a standard curve was plotted to calculate sample concentrations.

### Statistical analysis

Statistical analyses were conducted using GraphPad Prism (version 8, Inc., La Jolla, CA, USA). Data are presented as means ± SD. Comparisons among multiple groups were assessed using one-way analysis of variance (ANOVA) with Bonferroni post hoc test, while differences between two groups were evaluated using Student’s t-test. A p-value < 0.05 was considered statistically significant. Each experiment was performed with at least three biological replicates.

## Results

### *Abca1* identified as a key regulator of lipid metabolism post-SCI through mRNA sequencing and transcriptomics analysis

**Fig. 1:**
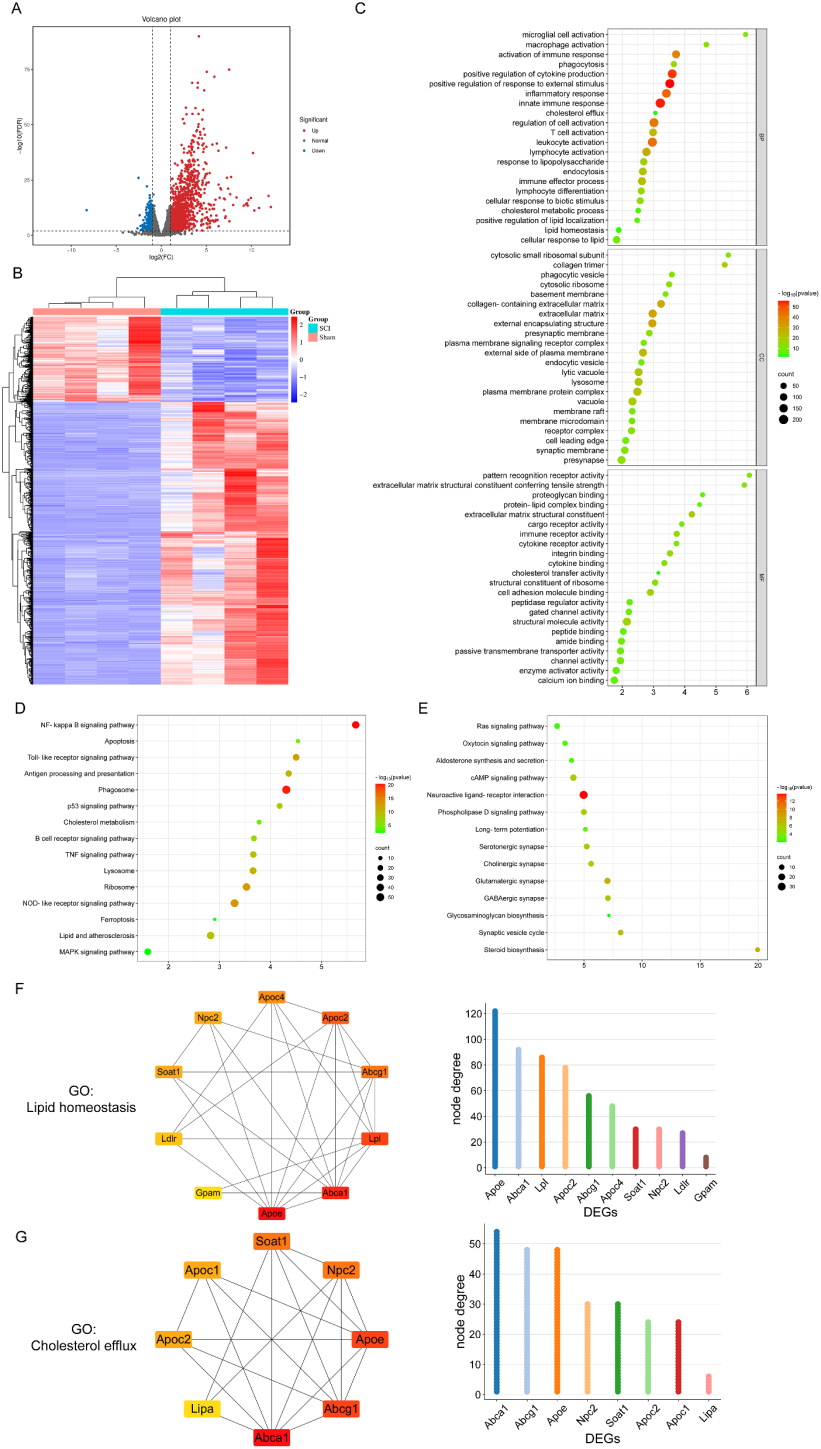
Identification and Bioinformatics analysis of DEGs between SCI and normal control tissues. **(A)** The valcano plot of DEGs. **(B)** The heatmap of the DEGs. **(C)** GO analysis of all DEGs. **(D)** KEGG analysis of up-regulated DEGs. **(E)** KEGG analysis of down-regulated DEGs. **(F)** PPI network of DEGs in lipid homeostasis pathway. **(G)** PPI network of DEGs in cholesterol efflux pathway. FDR < 0.01. *Abbreviations* SCI: spinal cord injury; DEGs: differentially expressed genes; BP, biological process; CC, cell component; MF, molecular function; GO, Gene Ontology; KEGG, Kyoto Encyclopedia of Genes and Genomes; PPI, Protein–protein interaction

A comparative molecular expression analysis was conducted using bioinformatics between SCI and normal spinal cord tissues to elucidate the altered mRNA expression following SCI. mRNA sequencing was performed on spinal cord samples from both Sham and SCI mice (14 days post-injury, dpi), and transcriptomic expression profiles were analyzed. The 14 dpi time point was chosen to capture the chronic phase of the injury response, where microglia are actively involved in tissue remodeling and repair processes. This allows for the study of sustained effects and the roles of microglia in the later stages of injury, providing insights into their functions during ongoing repair, inflammation resolution, and scar formation. Figure S1 illustrates the characteristics of sham and SCI spinal cord samples. The processed data boxplot indicates that the median for each sample is nearly on the same line (Figure S1A). The PCA plot demonstrates a clear differentiation between sham and SCI groups (Figure S1B). A total of 15,222 mRNAs were identified, and based on the set criteria of DEGs with a false discovery rate (FDR) < 0.01 and FC ≥ 2, significant DEGs between the two groups were observed. In comparison to the Sham group, the SCI group exhibited 2,042 DEGs, with 1,568 upregulated and 474 downregulated (Figure S1C). Initially, the results of DEGs were visually presented using a volcano plot (Fig. 1A). Subsequently, a global overview of all DEGs was visualized using K-means clustering and a heatmap (Fig. 1B). The clustering analysis demonstrated a clear separation of DEGs into two distinct groups for Sham and SCI, indicating good consistency among the four replicates.

Gene Ontology (GO) pathway enrichment analysis and Kyoto Encyclopedia of Genes and Genomes (KEGG) pathway enrichment analysis were conducted separately for upregulated and downregulated DEGs to comprehensively investigate the functional alterations of DEGs post-SCI. According to the GO analysis results, we observed significant enrichments in biological processes (BP) related to microglial cell activation, activation of the immune response, phagocytosis, positive regulation of cytokine production, inflammatory response, innate immune response, cholesterol efflux, response to lipopolysaccharide, cholesterol metabolic process, lipid homeostasis, cellular response to lipid, and macrophage activation, among others (Fig. 1F). Regarding cellular components (CC), the changes in DEGs were predominantly enriched in the cytosolic small ribosomal subunit, phagocytic vesicle, cytosolic ribosome, extracellular matrix, endocytic vesicle, membrane raft, and other cellular components (Fig. 1C). Changes in molecular functions (MF) were concentrated in extracellular matrix structural constituent, cell adhesion molecule binding, structural molecule activity, immune receptor activity, integrin binding, cytokine binding, cargo receptor activity, protein-lipid complex binding, cholesterol transfer activity, and other functional aspects (Fig. 1D). Based on the KEGG pathway analysis results, upregulated DEGs were primarily involved in pathways such as NF-kappa B signaling, apoptosis, Toll-like receptor (TLR) signaling, antigen processing and presentation, phagosome, p53 signaling, cholesterol metabolism, TNF signaling, lysosome, ribosome, NOD-like receptor signaling, ferroptosis, lipid and atherosclerosis, among others (Fig. 1E). In contrast, downregulated DEGs were mainly associated with pathways like Steroid biosynthesis, Synaptic vesicle cycle, Phospholipase D signaling, Neuroactive ligand-receptor interaction, cAMP signaling, Ras signaling, and others (Fig. 1F).

It is noteworthy that genes associated with lipid metabolism, such as *Abca1*, *Apoc1*, *Apoc2*, *Apoe*, *Lipa*, *Lpl*, and *Npc2*, exhibited significant upregulation in expression at 14 days post- SCI. Among these genes, *Abca1* plays a crucial role in regulating cholesterol efflux and modulating the phenotype of macrophages post-SCI, *Apoe* contributes to protecting the blood-spinal cord barrier and reducing post-SCI inflammatory responses, while *Npc2* is involved in intracellular cholesterol metabolism, thereby reducing lipid accumulation in lysosomes.

To gain deeper insights into the characteristics of lipid metabolism disruption following SCI, we conducted a GO biological process enrichment analysis on these DEGs. The results, visualized through a chord diagram (Figure S2), further confirm the pivotal role of lipid metabolism in the progression of SCI, aligning with previous research findings.

Additionally, a protein-protein interaction (PPI) network of these DEGs involved in lipid metabolism pathways was constructed using the STRING database (Fig. 1G/H). Further quantification of the importance of these genes within the network was performed using the cytoHubba application in Cytoscape (Fig. 1G/H). The results highlight the critical role of *Abca1* in pathways related to lipid homeostasis and cholesterol efflux.

In conclusion, our study suggests that lipid metabolism may play a crucial role in neuroinflammation and repair post-SCI. *Abca1* emerges as a key gene in lipid homeostasis and cholesterol efflux, indicating that addressing lipid metabolism disruption in microglia post-SCI could be a significant therapeutic target for SCI treatment.

### Lipid droplets accumulate in microglia at the injury center post-SCI

**Fig. 2:**
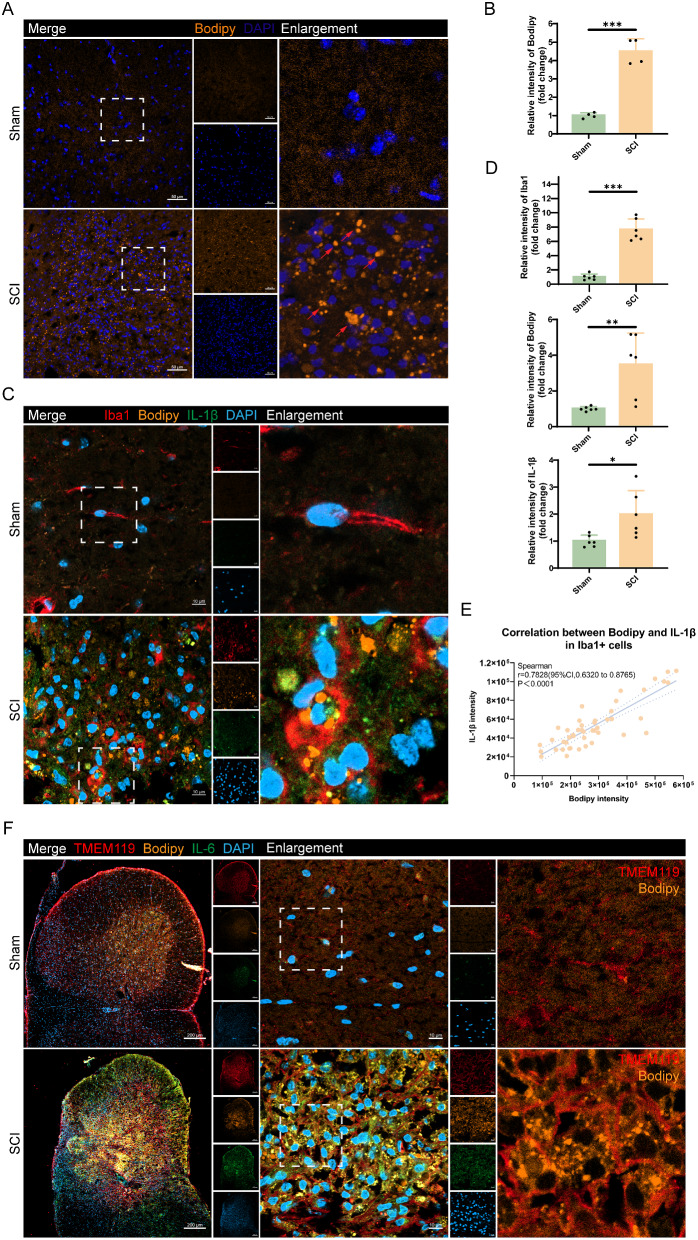
LDs accumulated in epicenter after SCI. **(A)** IF staining at 14 dpi, orange fluorescence for BODIPY, marks LDs; blue fluorescence for DAPI marks cell nuclei. Red arrows in the image denote LDs. Scale bar = 50 μm. **(B)** Relative intensity of BODIPY is significantly upregulated after SCI. **(C)** IF staining at 14 dpi, red fluorescence for Iba1 marks macrophage/microglia; orange fluorescence for BODIPY marks LDs; blue fluorescence for DAPI marks cell nuclei; green fluorescence for IL-1β. Scale bar = 10 μm. **(D)** The relative intensity of Iba1, BODIPY and IL-1β are significantly upregulated after SCI. **(E)** Correlation analysis shows a positive correlation between Bodipy and IL-1β. **(F)** IF staining at 14 dpi, red fluorescence for TMEM119 marks microglia; orange fluorescence for BODIPY marks LDs; blue fluorescence for DAPI marks cell nuclei; green fluorescence for IL-6. Scale bar = 10 μm. *P < 0.05; ** *P* < 0.01; ***P < 0.001

To observe and characterize the distribution of LDs at the injury epicenter in mice from the Sham and SCI groups (14 dpi), we employed our previously established SCI platform. The cervical spinal cord hemi-contusion injury model was induced at the C5 spinal cord segment in mice, followed by IF staining of spinal cord sections for morphological examination. BODIPY, a specific fluorescent dye for neutral lipids, was commonly used to detect LDs. The IF staining results revealed a significant increase in BODIPY fluorescence intensity at the injury epicenter in the SCI group compared to the Sham group (Fig. 2A, B). At the SCI epicenter, LDs exhibited larger sizes, higher numbers, and clustered accumulation, whereas LDs accumulation was nearly undetectable in the Sham group (Fig. 2A). This outcome indicates that LDs accumulated at the injury epicenter post-SCI.

To determine LDs accumulation in macrophage/microglia post-SCI, we performed BODIPY staining on spinal cord sections from both the Sham and SCI groups at 14 dpi. Co-IF staining was conducted using the microglia marker Iba1 and the inflammatory marker IL-1β. IF staining results demonstrated a noticeable increase in the fluorescence intensities of Iba1, BODIPY, and IL-1β at the injury epicenter 14 dpi compared to the Sham group. Interestingly, LDs primarily accumulated in Iba1^+^ cells at the injury epicenter post-SCI (Fig. 2C, D). Additionally, the LDs accumulation in Iba1^+^ cells post-SCI showed a positive correlation with the expression of the inflammatory marker IL-1β (Fig. 2E).

TMEM119 is a cell surface protein and a specific marker for microglial subpopulations in bone marrow and neural cells [37]. TMEM119 co-expresses with Iba1 in the branching and morphologically transformed microglia, playing a crucial role in identifying microglia and distinguishing them from infiltrating macrophages and other cell types. To further differentiate microglia and macrophages in the injury epicenter, we performed Co-IF of spinal cord sections from Sham and SCI groups at 14 dpi using BODIPY, TMEM119, and IL-6. We found that the injury center in our cervical spinal cord hemicontusion model is located in the gray matter. Compared to the Sham group, IF staining results showed significantly increased fluorescence intensity of TMEM119, BODIPY, and IL-6 at the injury epicenter at 14 dpi in the SCI group. Moreover, LDs in TMEM119^+^ cells were larger and more abundant in the SCI group, and IL-6 expression in TMEM119^+^ cells was significantly higher compared to the Sham group (Fig. 2F).

### DEMs identified and screened in C5 Hemicontusion SCI model

For the identification of critical miRNAs involved in post-SCI processes, we conducted small RNA sequencing using spinal cord specimens from both Sham and SCI groups (14 dpi). Subsequently, all low-quality reads, poly-N sequences, and sequences measuring less than 18 nt or greater than 30 nt were removed from the respective raw reads. After filtering out rRNA, tRNA, and snoRNA, the remaining unannotated reads were utilized to identify miRNAs. These reads were aligned against the mature sequences of known miRNAs from the miRBase (v22) database, allowing for a maximum of one mismatch within the upstream 2 nt and downstream 5 nt range. The identified reads were considered known miRNAs. In total, 1443 known miRNAs were identified, and 1370 miRNAs met the filtering criteria, which were retained for subsequent differential analysis.

**Fig. 3:**
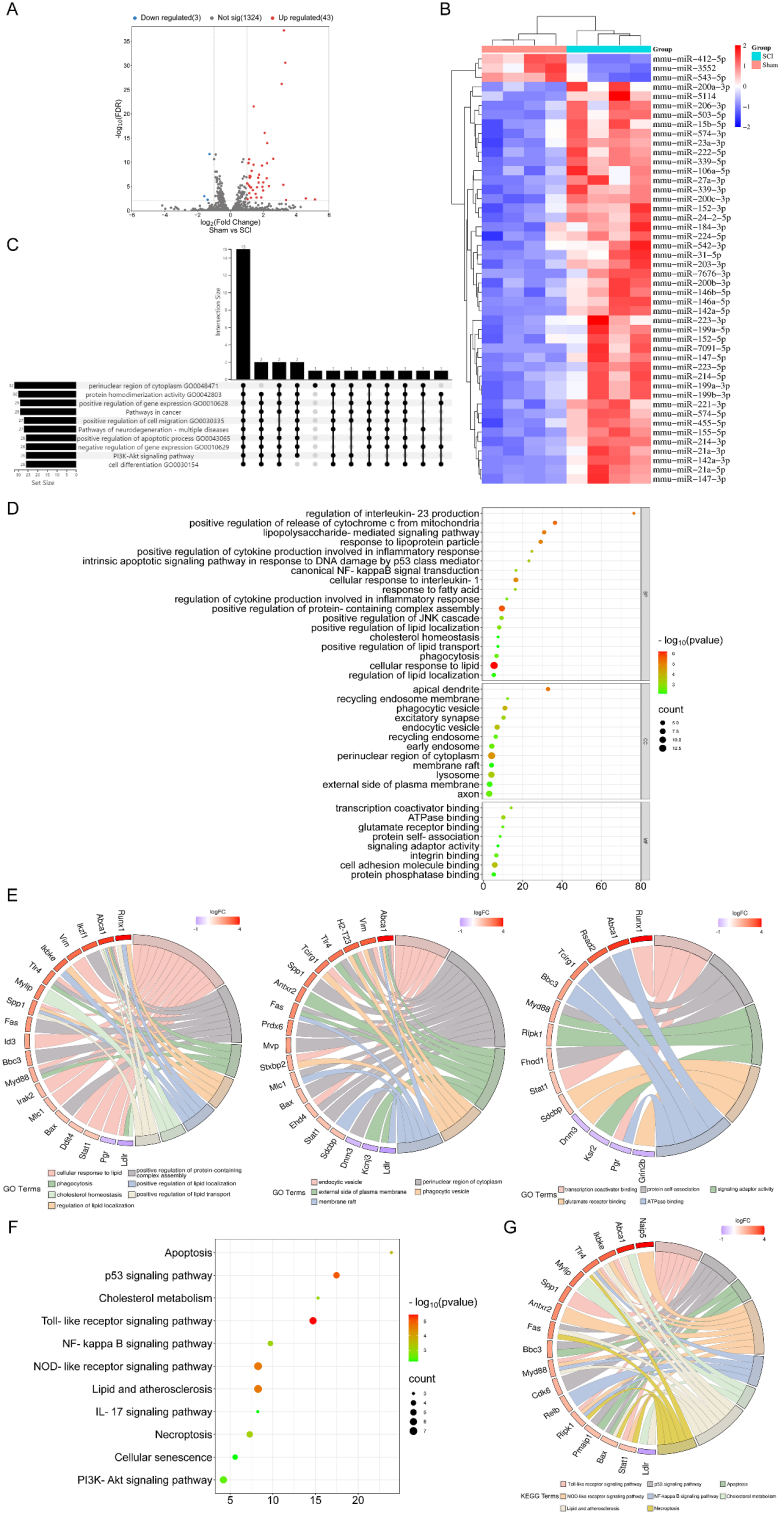
sRNA sequencing and GO/KEGG pathway enrichment analysis of DEMs. **(A)** The valcano plot of DEMs. **(B)** The heatmap of the DEMs. **(C)** Upset plot was used to display the enriched GO and KEGG pathways, sorted by the number of enriched entries. **(D)** The enriched GO terms of DEGs in the network; **(E)** The relationship between enriched mRNAs in GO pathways; **(F)** The KEGG pathway enrichment analysis of mRNAs in the network; **(G)** The relationship between enriched mRNAs in KEGG pathways; FDR < 0.01. *Abbreviations* DEMs: differentially expressed miRNAs

To elucidate the expression patterns of miRNAs post-SCI, we performed a differential expression analysis comparing miRNAs between the Sham and SCI groups. The criteria set for identifying DEMs were a P-value < 0.01 and a FC ≥ 2. The results revealed 46 DEMs between the Sham and SCI groups (14 dpi), comprising of 43 upregulated DEMs and 3 downregulated DEMs (Fig. 3A). A volcano plot was employed to visually represent the expression distribution of DEMs between the groups (Fig. 3B), and K-means clustering along with a heatmap provided a comprehensive overview of all DEMs (Fig. 3C). The clustering analysis demonstrated a distinct separation of known DEMs between the Sham and SCI groups, indicating excellent consistency among the four replicates.

In order to gain deeper insights into the regulatory functions of DEMs post-SCI, we utilized the miEAA 2.1 analysis platform to conduct GO and KEGG pathway analyses for DEMs target genes. The GO analysis indicated that DEMs were found to be primarily associated with pathways such as intracellular receptor signaling, positive regulation of Notch signaling, negative regulation of cell death, SMAD protein signal transduction, cell differentiation, integrin binding, positive regulation of cholesterol efflux, leukocyte activation involved in inflammatory response, lipopolysaccharide-mediated signaling, positive regulation of JNK cascade, positive regulation of apoptotic process, monocyte chemotaxis, response to xenobiotic stimulus, among others (Figure S3). The KEGG analysis revealed that DEMs target genes were involved in signaling pathways including pathways of neurodegeneration, PI3K-Akt signaling, Alzheimer’s disease, MAPK signaling, Ras signaling, cAMP signaling, lipid and atherosclerosis, TNF signaling, ErbB signaling, and others (Figure S3). These results suggest that miRNAs may exert regulatory effects post-SCI by targeting genes within these pathways.

To identify miRNAs with broad effects post-SCI, we employed an Upset plot to visualize enriched GO and KEGG pathways, sorted by the number of enriched entries (Fig. 3D). Among these, 15 DEMs, including miR-106a-5p, miR-146a-5p, miR-152-3p, miR-155-5p, miR-199a-3p, miR-200a-3p, miR-200b-3p, miR-203-3p, miR-206-3p, miR-21a-5p, miR-221-3p, miR-223-3p, miR-23a-3p, miR-27a-3p, miR-31-5p, exhibited enrichment across various GO and KEGG pathways. Table S1 provides the FDR values and log2FC for these 15 DEMs.

To further investigate the functions of these 15 DEMs post-SCI, we constructed a miRNA-mRNA regulatory network encompassing 85 mRNAs and 15 miRNAs, providing a detailed depiction of the interactions between DEMs and DEGs. This network facilitates a better understanding of the role of miRNAs in SCI (Figure S4A).

GO analysis of the regulatory network on mRNA revealed significant enrichment in Biological Processes (BP) terms, such as regulation of interleukin-23 production, lipopolysaccharide-mediated signaling pathway, response to lipoprotein particle, canonical NF-kappaB signal transduction, cellular response to interleukin-1, positive regulation of JNK cascade, positive regulation of lipid localization, cholesterol homeostasis, positive regulation of lipid transport, phagocytosis, cellular response to lipid, and regulation of lipid localization. Cellular Component (CC) terms included apical dendrite, recycling endosome membrane, phagocytic vesicle, excitatory synapse, endocytic vesicle, perinuclear region of cytoplasm, and lysosome. Molecular Function (MF) terms comprised transcription coactivator binding, ATPase binding, glutamate receptor binding, protein self-association, and signaling adaptor activity (Fig. 3D). Figure 3E illustrates the relationships between mRNA and enriched pathways, while Fig. 3F highlights the top 11 most significant KEGG pathways. These pathways, including apoptosis, p53 signaling pathway, cholesterol metabolism, TLR signaling pathway, NF-kappa B signaling pathway, lipid and atherosclerosis, NOD-like receptor signaling pathway, IL-17 signaling pathway, necroptosis, cellular senescence, and PI3K-Akt signaling pathway, are implicated in the pathological development of SCI. Furthermore, Fig. 3G delineates the relationships between mRNA and the enriched KEGG pathways.

### miR-223 identified as a key regulator of lipid homeostasis post-SCI via *Abca1*

**Fig. 4:**
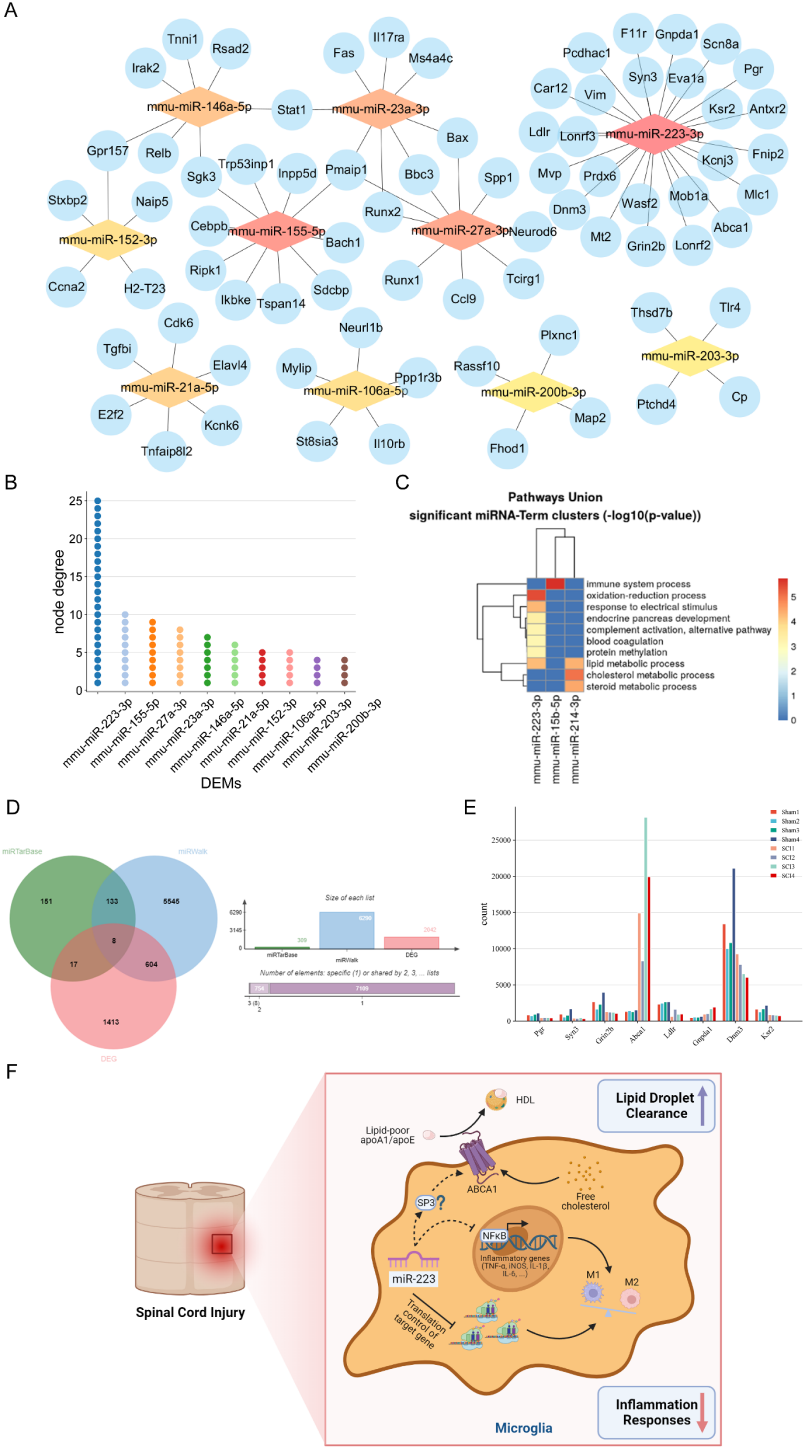
Reconstruction of miRNA-mRNA network. **(A)** The miRNA-mRNA network. Node degree were exhibited by the color. miRNAs are represented by a diamond shape, and mRNAs are represented by a circle shape; **(B)** Node degree of DEMs in miRNA-mRNA network; **(C)** Pathways Union enrichment analysis on miR-223-3p; **(D)** Venn diagrams through the miRTarBase and miRWalk websites to predict target genes of miR-223, intersecting them with DEGs; **(E)** Expression patterns of intersecting genes; **(F)** miR-223 suppresses inflammation in microglia by facilitating LDs clearance

The CytoHubba application in Cytoscape software was utilized to calculate node degrees within the regulatory network. The top 10 nodes, including miR-223-3p, miR-155-5p, miR-27a-3p, miR-23a-3p, miR-146a-5p, miR-21a-5p, miR-152-3p, miR-106a-5p, miR-200b-3p, and miR-203-3p, were identified as hub nodes and visualized using Cytoscape software (Fig. 4A/B).

In the aforementioned miRNA-mRNA regulatory network, miR-223-3p demonstrated the highest node degree, suggesting its potential key role in the development of SCI. Nguyen et al. previously identified miR-223 as a global regulator of RNA translation in cholesterol efflux and inflammatory pathways in macrophages, preventing atherosclerosis [38]. However, limited knowledge exists regarding the function of miR-223 in SCI, prompting our focused investigation into miR-223.

Pathways Union enrichment analysis on miR-223-3p, conducted via DIANA mirPath v.4, revealed significant enrichment of miR-223-3p in biological processes such as oxidative-reduction processes, complement activation, blood coagulation, protein methylation, and lipid metabolic process (Fig. 4C). Given the interplay between lipid metabolism and inflammation in SCI pathogenesis, we aimed to elucidate the potential mechanisms of miR-223 action in SCI. Therefore, we employed Venn diagrams through the miRTarBase and miRWalk websites to predict target genes of miR-223, intersecting them with DEGs, which resulting in 8 intersecting genes (Fig. 4D). Table S2 lists these intersecting genes along with their full names and descriptions, while Fig. 4E illustrates the expression patterns of these intersecting genes across the sample groups. Among them, miR-223 potentially participates in lipid metabolism by regulating key genes, such as *Abca1*, which involved in lipid homeostasis and cholesterol efflux. However, further research is required to determine whether miR-223 can regulate lipid metabolism disruption in microglia after SCI (Fig. 4F). Consequently, miR-223 and the BV2 microglial cell line were selected for further investigation.

### miR-223 facilitates ABCA1 expression, promotes myelin debris degradation and lipid droplets clearance in myelin debris-stimulated BV2

ABCA1 is a crucial lipid transport protein widely expressed in various cells, including microglia. Bioinformatics data suggest that miR-223 may regulate ABCA1 expression after SCI. Recent studies indicate that miR-223 directly targets and inhibits the transcription factor Specificity Protein 3 (*SP3*), indirectly promoting ABCA1 expression, and facilitating lipid efflux in macrophages [38, 39]. To verify whether miR-223 similarly regulates ABCA1 expression and lipid efflux in microglia, we utilized lentiviruses overexpressing miR-223 (miR-223-OE) and negative controls (miR-223-NC) to transfect BV2 cells. Subsequently, we induced lipid burden in BV2 cells using myelin debris.

**Fig. 5:**
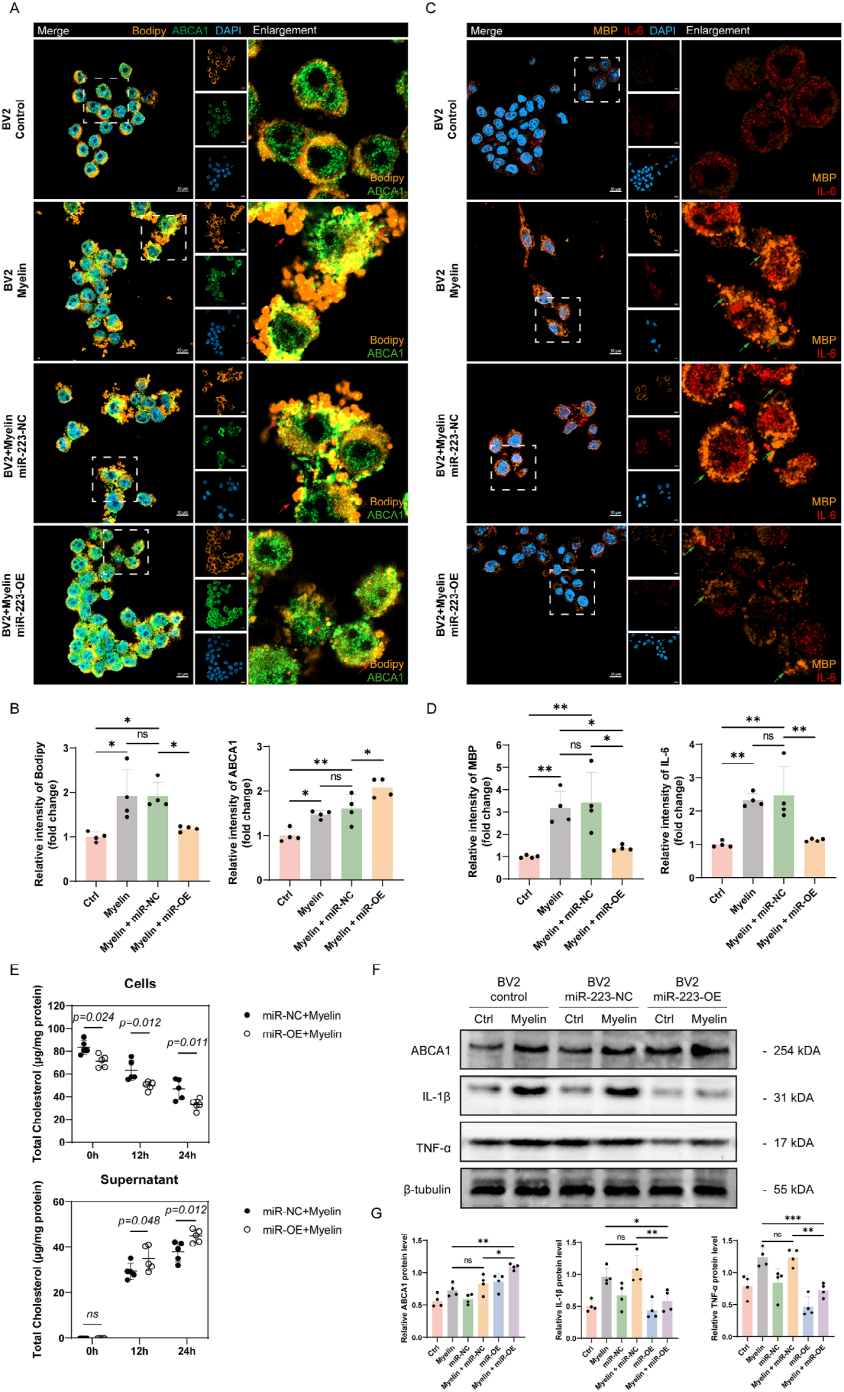
miR-223 induces the upregulation of ABCA1, reduces LDs accumulation, promotes myelin debris degradation and enhances cholesterol efflux in myelin debris triggered-BV2. **(A)** IF staining of LDs (BODIPY, orange) and ABCA1 (green) in the afore-mentioned 4 groups. **(B)** Quantification analysis of the mean fluorescence intensity (MFI) of Bodipy and ABCA1 (*n* = 4). **(C)** IF staining of myelin debris (MBP, orange) and IL-6 (red) in the afore-mentioned 4 groups. **(D)** Quantification analysis of the MFI of MBP and IL-6 (*n* = 4). **(E)** Quantitative analysis of total cholesterol content in BV2 cells and supernatant (*n* = 5). **(F/G)** Quantitative analysis of ABCA1, IL-1β and TNF-α expression in BV2 cells using Western blot analysis normalized with the housekeeping protein β-tubulin in the six groups mentioned above (*n* = 4). Scale bars, 10 μm. ns: no significance, *P < 0.05, **P < 0.01, ***P < 0.001

We assessed ABCA1 expression using immunofluorescence (IF) and labeled LDs in BV2 cells from each group with Bodipy. After 24 h of myelin debris treatment, both ABCA1 and Bodipy fluorescence intensities significantly increased in BV2 cells. Importantly, compared to the miR-223-NC group, the miR-223-OE group exhibited markedly higher ABCA1 fluorescence intensity and lower LDs levels (Fig. 5A/B). Subsequently, we labeled myelin debris in BV2 cells from each group with MBP and evaluated BV2 cell inflammation using IL-6. IF staining revealed significant accumulation of MBP and the inflammatory marker IL-6 in BV2 cells after 24 h of myelin debris treatment. However, compared to the miR-223-NC group, the miR-223-OE group showed significantly lower levels of MBP and IL-6 (Fig. 5C/D).

In addition, we used assay kits to measure cholesterol levels in BV2 cells and their supernatants at 0, 12, and 24 h after myelin debris treatment to investigate the impact of miR-223 on cholesterol efflux in microglia. The results showed a significant increase in cholesterol levels in BV2 cells shortly after myelin debris treatment, which subsequently decreased over time. Conversely, cholesterol levels in the supernatants gradually increased. Nevertheless, the miR-223-OE group exhibited significantly lower intracellular cholesterol levels compared to the miR-223-NC group, while cholesterol levels in the supernatants were significantly higher than those in the miR-223-NC group (Fig. 5E).

Additionally, Western blot analysis corroborated the findings from immunofluorescence, showing a significant increase in ABCA1, IL-1β, and TNF-α expression after myelin debris treatment compared to the control group. Furthermore, ABCA1 expression in the miR-223-OE group was significantly higher than in the miR-223-NC group, while IL-1β and TNF-α expression in the miR-223-OE group was significantly lower than in the miR-223-NC group (Fig. 5F/G).

In summary, myelin debris triggers the accumulation of LDs and enhances ABCA1 expression, suggesting that increased ABCA1 expression serves as a negative feedback regulation mechanism against lipid accumulation. This may be crucial for maintaining lipid metabolism homeostasis in microglia, enabling them to process large quantities of myelin debris. miR-223 can further enhance ABCA1 expression in BV2 cells, thereby promoting LDs clearance and cholesterol efflux. Additionally, miR-223 facilitates the degradation of myelin debris in BV2 cells and suppresses the expression of inflammatory cytokines induced by myelin debris.

### miR-223 facilitates ABCA1 expression and promotes lipid droplets clearance in LPS-stimulated BV2

Foamy microglia exhibit altered migration and phagocytic capabilities, promoting a pro-inflammatory phenotype. Regulating the expression of foamy microglia lipid metabolism-related genes can modulate lipid metabolism disorders and affect their inflammatory phenotype, thereby further influencing motor function recovery in mice following SCI. Therefore, there is a crucial link between foamy microglial lipid metabolism and the inflammatory phenotype. Marschallinger et al. recently confirmed in their study that LPS induces LDAM formation [13]. Subsequently, we induced M1-type inflammatory activation in BV2 cells using LPS.

We assessed ABCA1 expression using IF, observing a significant increase in ABCA1 fluorescence intensity in BV2 cells after 24 h of LPS treatment. Notably, the miR-223-OE group exhibited significantly higher ABCA1 fluorescence intensity compared to the miR-223-NC group. Subsequently, we labeled LDs in BV2 cells of each group using Bodipy and conducted IF staining. The results revealed a noticeable accumulation of LDs in BV2 cells after 24 h of LPS treatment. However, the miR-223-OE group exhibited significantly lower levels of LDs compared to the miR-223-NC group (Figure S5).

Additionally, we measured cholesterol levels in BV2 cells and cell culture supernatants at 0, 12, and 24 h after LPS treatment using a commercial kit to investigate the impact of miR-223 on cholesterol efflux in LPS-activated microglia. The results demonstrated a significant increase in cholesterol levels in BV2 cells shortly after LPS treatment, followed by a gradual decline. Conversely, cholesterol levels in the cell culture supernatants increased progressively. Additionally, intracellular cholesterol levels were significantly lower in the miR-223-OE group compared to the miR-223-NC group, whereas cholesterol levels in the cell culture supernatants were significantly higher in the miR-223-OE group compared to the miR-223-NC group (Figure S5C).

Furthermore, Western blot analysis consistent with the IF results, showing an increasing of ABCA1 expression after LPS treatment compared to the control group. Moreover, the miR-223-OE group exhibited a significantly higher expression of ABCA1 compared to the miR-223-NC group (Figure S5D/E).

In summary, consistent with myelin debris, LPS promotes LDs accumulation while also enhancing ABCA1 expression in BV2 cells. Furthermore, miR-223 further upregulates ABCA1 expression in LPS-stimulated BV2 cells, thereby facilitating LDs clearance and cholesterol efflux.

### miR-223 suppresses inflammatory activation and promotes anti-inflammatory polarization in microglia

**Fig. 6:**
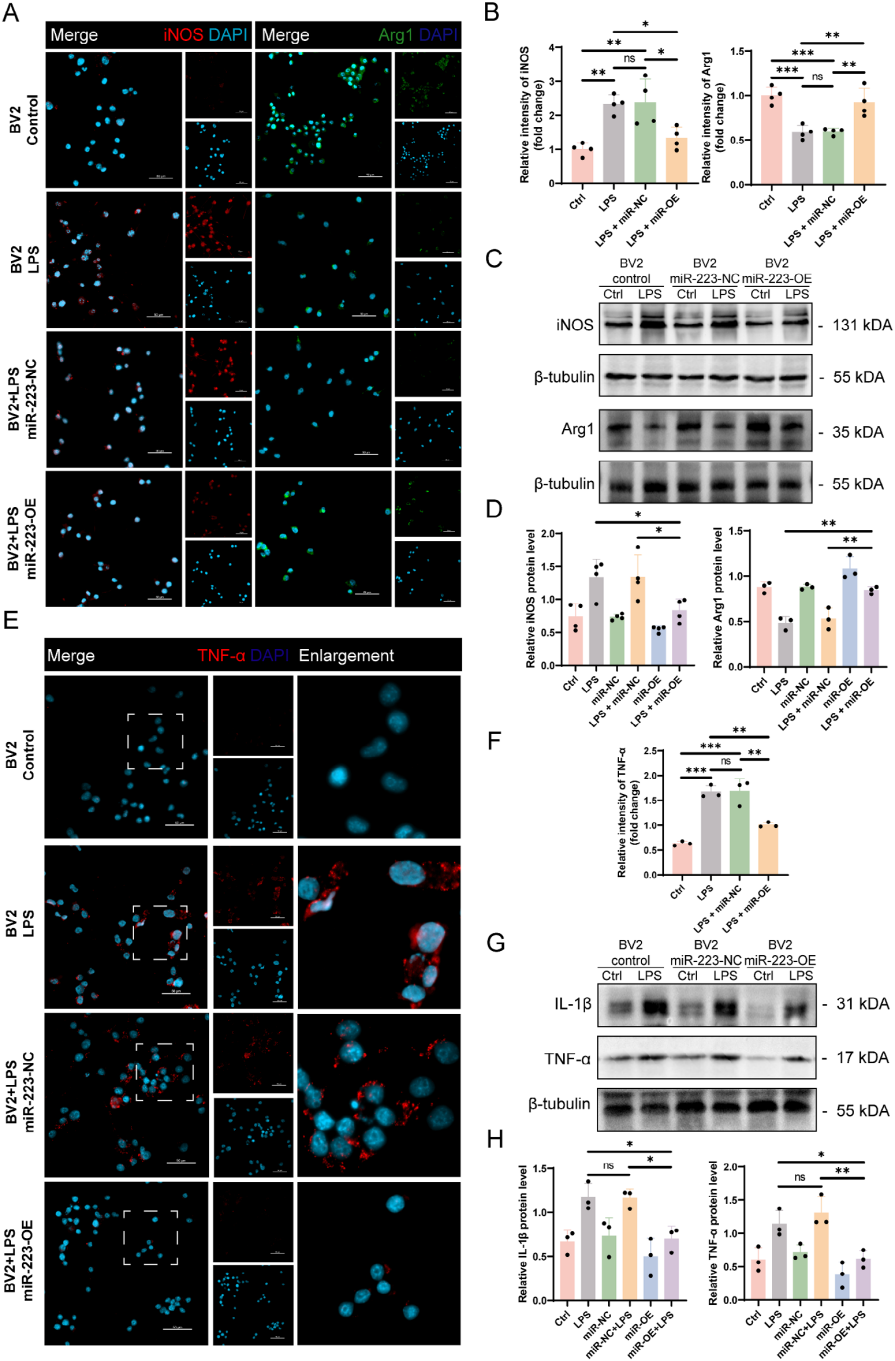
miR-223 regulates the polarization phenotype and inflammatory factor levels of BV2. **(A)** IF staining of M1 polarization of BV2 (iNOS, red) and M2 polarization of BV2 (Arg1, green) in the afore-mentioned 4 groups. **(B)** Quantitative analysis of MFI of iNOS and Arg1 (*n* = 4). **(C/D) **Quantitative analysis of iNOS and Arg1 expression in BV2 using Western blot analysis normalized with the housekeeping protein β-tubulin in the six groups mentioned above (*n* ≥ 3). **(E)** IF staining of inflammatory factor (TNF-α, red) in BV2 in the afore-mentioned 4 groups (*n* = 4). **(F)** Quantitative analysis of MFI of TNF-α (*n* = 3). **(G/H)** Quantitative analysis of inflammatory factor IL-1β and TNF-α expression in BV2 using Western blot analysis normalized with the housekeeping protein β-tubulin in the same six groups (*n* = 3). ns: no significance, *P < 0.05, **P < 0.01, ***P < 0.001

To gain deeper insights into the regulatory role of miR-223 in microglial inflammation, we induced M1-type inflammatory activation in BV2 cells by treating them with LPS for 24 h. Subsequently, we analyzed the expression levels of M1 polarization marker iNOS and the M2 polarization marker Arg1 through Western blot and IF in different groups. Compared to the control group, the expression of iNOS significantly increased, while the expression of Arg1 markedly decreased in BV2 cells after 24 h of LPS stimulation. However, in LPS-stimulated BV2 cells, miR-223-OE led to a significant reduction in iNOS expression and a simultaneous increase in Arg1 expression compared to BV2 cells in the miR-223-NC group (Fig. 6A-D). These observations indicate that miR-223 can inhibit M1-type pro-inflammatory activation in microglia and promote a polarization towards the anti-inflammatory M2 phenotype in vitro, consistent with previous research findings.

Thereafter, we examined the expression levels of several classical pro-inflammatory cytokines in the different groups through Western blot and IF. After 24 h of LPS stimulation, the miR-223-OE BV2 cells exhibited lower levels of IL-1β and TNF-α compared to the miR-223-NC BV2 cells, indicating the potential anti-inflammatory properties of miR-223 (Fig. 6E-H).

In summary, miR-223 mitigates LPS-induced inflammation in BV2 cells. Its mechanism of action may involve alleviating LDs accumulation in BV2 cells and promoting polarization towards the M2 anti-inflammatory state. Therefore, we hypothesize that miR-223 may play a neuroprotective role in the progression of SCI by mitigating LDs accumulation in microglia.

## Discussion

LDAM represent a recently identified state of microglia characterized by defective phagocytic function, elevated ROS production, and extensive release of inflammatory factors [13]. Our transcriptomic analysis at the epicenter of SCI in mice indicated disrupted lipid metabolism, which was further confirmed by IF staining showing significant accumulation of LDs within microglia. Through integrated miRNA and mRNA multi-omics analysis on spinal cord tissues from SCI mice, we identified the pivotal role of miR-223 in post-SCI lipid metabolism regulation. miR-223, a key gene in regulating lipid homeostasis and promoting cholesterol efflux, participates in lipid metabolism by modulating the expression of ABCA1. Furthermore, in vitro experiments demonstrated that the upregulation of miR-223 promotes the expression of ABCA1, a lipid transport membrane protein, while reducing lipid load in BV2 cells. It facilitates myelin debris degradation and induces polarization towards an anti-inflammatory M2 phenotype. These findings suggest that the upregulation of miR-223 can enhance ABCA1 expression, consequently facilitating LDs clearance and suppressing the inflammatory response in microglia.

Microglia, recognized as resident phagocytic cells of the innate immune system [40], stand as the first responders to various central nervous system injuries. Following SCI, the clearance of myelin debris and cellular fragments is orchestrated by microglia, aiming to establish an environment conducive to regeneration [41]. We employed high-throughput sequencing techniques to sequence the mRNA and miRNA expression profiles during subacute phase of SCI in our mouse model, analyzing the characteristics and expression levels of DEGs and DEMs. Bioinformatics analysis unveiled that DEGs in mice after SCI are functionally enriched in GO terms such as microglial cell activation, activation of the immune response, inflammatory response, cholesterol efflux, and lipid homeostasis. The pathological aggregation and proliferation of various cells lead to a microenvironment filled with various mediators, including pro-inflammatory chemokines/cytokines and ROS [42, 43].

While microglia are essential for myelin debris clearance, excessive myelin uptake can lead to the formation of “foamy microglia” [44], which ultimately contribute to secondary tissue damage in SCI. LDs are cellular organelles storing various lipid and protein components, and play a central role in cellular metabolism and lipid homeostasis [45]. Our study observed a substantial activation of microglia and evident LDs accumulation at the SCI epicenter in mice. We employed IL-1β and IL-6, classical inflammatory factors, to assess microglial inflammation and found a positive correlation between LDs accumulation in microglia at the SCI epicenter and the expression levels of IL-1β, suggesting a relationship between lipid load and inflammation. Recent studies in aged mouse brain microglia have identified a phenomenon called LDAM, characterized by severe phagocytic defects, associated with increased ROS and pro-inflammatory cytokine release [13]. Similarly, LDs accumulation has been observed in microglia from patients with Alzheimer’s and Parkinson’s diseases [46], highlighting the potential involvement of lipid metabolism disruption in the onset and progression of neurological disorders. Lipid overload in microglia may trigger adverse immune responses, exacerbating secondary damage. Therefore, we hypothesize that reducing lipid overload in microglia could contribute to alleviating or even reversing their inflammatory phenotype in SCI.

Macrophage cholesterol efflux, facilitated by ABCA1, plays a pivotal role in alleviating lipid accumulation and atherosclerosis development, making ABCA1 and macrophage cholesterol efflux as primary targets for preventing and treating atherosclerosis [47]. In our study, transcriptomic analysis identified *Abca1* as a key gene in regulating lipid homeostasis and promoting cholesterol efflux, suggesting a potentially significant role in the intricate regulatory network of SCI. RNA sequencing results revealed a substantial upregulation of *Abca1* expression at the SCI epicenter in mice. ABCA1, identified as a lipid pump, facilitates the extracellular removal of cholesterol and phospholipids [48]. ABCA1 in microglia influences LPS-induced microglial activation, specifically buffering microglia activation and promoting an anti-inflammatory response [49]. In peripheral tissues, ABCA1-mediated Apolipoprotein A-I lipidation is crucial HDL generation, which is known for its recognized anti-inflammatory properties [50]. ABCA1 may impact microglial cell activation by influencing cellular lipid metabolism. In cultured microglia lacking ABCA1, reduced cholesterol efflux to lipoprotein receptors (including Apolipoprotein E) and increased lipid content were observed [51]. Additionally, ABCA1 activity may affect TLR signaling in microglia. TLRs, as pattern recognition receptors, recognized pathogen-associated molecules (such as LPS, endogenous molecules, and heat shock proteins released by dead cells) [52, 53], associated with neuronal injury and neurodegenerative changes. It has been reported that in multiple sclerosis, myelin-induced elevation of Stearoyl-CoA desaturase-1 (SCD1) levels leads to a reduction in ABCA1 cell surface expression, affecting cholesterol efflux and promoting lipid accumulation. This, in turn, results in an inflammatory phagocyte phenotype [54]. ABCA1-deficient microglia/macrophages show increased TLR4 cell surface expression and signalling, leading to enhanced NF-κB activation and an increased release of TNFα and other pro-inflammatory mediators [55]. Above all, these findings elucidate the relationship between ABCA1 and neuroinflammation, suggesting that upregulating ABCA1 holds great promise for inhibiting microglial lipid loading and inflammation in SCI therapy.

miRNAs are small non-coding RNAs critical for post-transcriptional gene regulation and are recognized as essential regulatory factors in the pathobiology of diseases, exerting a pivotal influence on disease onset and progression. miRNAs are closely associated with many significant diseases. The advancement and cost reduction of gene sequencing technologies, along with the development of RNA modification techniques and breakthroughs in delivery systems, have made miRNAs a focal point in disease treatment [56, 57]. Employing the miEAA 2.1 analysis platform, we screened DEMs with extensive regulatory effects after SCI and conducted a comprehensive multi-omics analysis. Our findings highlight the significant role of miR-223 in lipid metabolism and inflammation regulation following SCI. We conducted Pathways Union enrichment analysis on miR-223 using the DIANA mirPath v.4 analysis platform, revealed a substantial enrichment of miR-223 in biological processes such as redox processes, complement activation, coagulation processes, protein methylation, and lipid metabolism. The miR-223 coding gene, located at the q12 locus of the X chromosome, exhibits a highly conserved sequence [58], suggesting its potential involvement in crucial physiological events. Originally discovered in the hematopoietic system, miR-223 has been implicated in cellular differentiation and inflammatory responses, participating in the regulation of myeloid cell differentiation, neutrophil activation, and macrophage activation [59]. miR-223 exerts control over cellular cholesterol levels by directly inhibiting genes involved in cholesterol biosynthesis and uptake in hepatocytes and coronary artery endothelial cells [39, 60]. These studies collectively affirm the critical role of miR-223 in lipid metabolism and inflammation in vitro. In the nervous system, miR-223 not only inhibits neuroinflammation but also promotes repair processes [61, 62]. By suppressing the M1 polarization of microglia, miR-223 alleviates pro-inflammatory reactions and reduced brain ischemia-reperfusion injury [63]. miR-223 is indispensable for efficient M2-related phenotypes and functions, including phagocytic activity, which facilitates the effective clearance of myelin debris and promotes remyelination [62].

Our study unveils miR-223 as a pivotal regulator of microglial polarization. In vitro experiments demonstrated that miR-223 overexpression inhibited the activation of BV2 cells toward a pro-inflammatory phenotype (M1), while promoting their shift toward an anti-inflammatory phenotype (M2). This phenomenon correlated with decreased expression of the M1 marker iNOS and increased expression of the M2 marker Arg1. Consistent with previous observations, miR-223 overexpression in BV2 cells suppressed the expression of the inflammatory cytokines IL-1β and TNF-α. Indeed, our findings align with earlier observations indicating that reduced miR-223 expression in mice on a high-fat diet exacerbates obesity-related adipose tissue inflammation through the enhancement of classical pro-inflammatory responses [64]. The absence of miR-223 induces macrophages toward a pro-inflammatory phenotype (M1 activation) [65]. Within macrophages, miR-223 modulates the expression of IL-1β by targeting Nlrp3 [66]. In this context, we identify miR-223’s ability to enhance ABCA1 expression in microglia, thus regulating lipid homeostasis and concurrently controlling pro-inflammatory responses. This further underscores the crucial role of miR-223 in LDs clearance and inflammation regulation in microglia. This discovery opens new avenues for future research in the treatment of SCI.

However, the regulatory role of miR-223 in microglial lipid homeostasis in the context of SCI has not been reported. Through target gene prediction using miRTarBase and miRWalk, we identified miR-223 as a crucial regulator of ABCA1 following SCI. Previous research has highlighted several miRNAs, such as miR-33, miR-144, miR-148a, and miR-302a, which modulate lipid metabolism. However, most miRNAs typically hinder lipid clearance by inhibiting ABCA1 expression directly. In contrast, miR-223 stands out as one of the few miRNAs capable of indirectly activating *Abca1*, promoting lipid efflux. Previous studies have indicated that the promotive effect of miR-223 on *Abca1* relies on the inhibition of *Sp3* [38, 39]. Therefore, upregulating miR-223 expression emerges as a feasible strategy to enhance ABCA1 expression, reduce cellular lipid burden, and suppress inflammatory responses. We induced BV2 cell line with myelin debris to engulf myelin fragments, and in line with other research groups, we used LPS to induce LDs accumulation in the BV2 cells, thereby creating an LDAM cellular model [13]. We observed a significant increase in LDs levels and ABCA1 expression in BV2 cells following induction with myelin debris or LPS, which we attribute to a negative feedback regulation induced by lipid accumulation that leads to an increase in ABCA1 expression. Indeed, ABCA1 expression is influenced by intracellular lipid levels, and to counteract lipid overload, microglia maximize ABCA1 activity to facilitate lipid efflux. Therefore, further upregulating ABCA1 expression appears to be a viable approach to promote LDs clearance and reverse the LDAM cell subtype. Encouragingly, our study discovered that miR-223 promotes the clearance of LDs by upregulating ABCA1 expression in BV2 cells, while also enhancing the degradation of intracellular myelin debris. Importantly, miR-223 can independently promote ABCA1 expression and LDs clearance regardless of the microglial polarization state. By upregulating miR-223 expression, we alleviated LDs accumulation in BV2 cells, a significant effect observed regardless of whether the microglia were induced by LPS activation. The upregulation of miR-223 expression may be a potential mechanism to restore lipid balance and reduce inflammation in damaged microglia by inhibiting inflammatory pathways and maximizing ABCA1 activity and anti-inflammatory signals. This discovery provides new insights into the regulation mechanisms of inflammation after SCI.

This study has several limitations that should be acknowledged. Firstly, we utilized bioinformatics analysis to predict* Abca1* as a target gene of miR-223, and our experimental results in concordance with other research findings revealed that miR-223 positively regulates the expression of ABCA1, These studies suggest that miR-223 may promote ABCA1 expression through various indirect mechanisms, with the most extensively studied being miR-223’s inhibition of its direct target gene *Sp3*, thereby indirectly promoting ABCA1 expression. Due to our experiments focused on the phenomenon of miR-223 overexpression leading to ABCA1 upregulation, further investigation is needed to determine whether the promotive effect of miR-223 on *Abca1* in microglia depends on the inhibition of its target transcription factor, *Sp3*. Secondly, we did not employ methods such as miR-223 knockdown to validate the opposing trends of ABCA1 expression, LDs clearance, or microglia inflammation. Thus, we only demonstrated the positive impact of miR-223 overexpression on ABCA1, LDs clearance, intracellular myelin debris degradation and microglia inflammation. Thirdly, although our in vitro experiments confirmed that miR-223 is a crucial component in regulating lipid homeostasis in microglia, a comprehensive understanding of its role in SCI requires validation through in vivo experiments in future studies.

## Conclusions

In conclusion, our study highlights, for the first time, the pivotal role of miR-223 in LDs regulation during SCI. Evidently, by promoting ABCA1 expression and facilitating LDs clearance, and enhancing the degradation of intracellular myelin debris, miR-223 exerts a critical regulatory influence on microglia, contributing to the attenuation of the pro-inflammatory phenotype and maintenance of lipid homeostasis observed in microglia after SCI. Consequently, the potential for systemic or microglia-specific delivery of miR-223 via viral vectors, nanoparticles, or extracellular vesicles to modulate ABCA1 and associated LDs clearance may emerge as a promising therapeutic strategy for treating SCI [67]. This discovery not only broadens our comprehension of the inflammatory mechanisms underlying SCI but also provides vital clues for the development of innovative therapeutic approaches.

### Electronic supplementary material

Below is the link to the electronic supplementary material.

Supplementary Material 1

Supplementary Material 2

## Data Availability

The data that support the findings of this study are available from the corresponding authors upon reasonable request.

## Abbreviations

SCI: Spinal cord injury
LDs: Lipid droplets
miRNA: microRNA
Abca1: ATP-binding cassette transporter A1
LDAM: Lipid-droplet-accumulating microglia
mRNA: messenger RNA
DEMs: Differentially expressed microRNAs
DEGs: Differentially expressed genes
IF: Immunofluorescence
FC: Fold change
GO: Gene ontology
KEGG: Kyoto encyclopedia of genes and genomes
LPS: Lipopolysaccharide
NC: Negative control
FDR: False discovery rate
BP: Biological processes
CC: Cellular components
MF: Molecular functions
PPI: Protein-protein interaction
SP3: Specificity protein 3
MFI: Mean fluorescence intensity
