# Supplementary material for: miR-223 accelerates lipid droplets clearance in microglia following spinal cord injury by upregulating ABCA1
Source: J Transl Med. 2024 Jul 15;22:659. doi: 10.1186/s12967-024-05480-5 (PMC11247820; doi:10.1186/s12967-024-05480-5)
Supplement: Supplementary file 1 — Supplementary Material 1 [file 12967_2024_5480_MOESM1_ESM.pdf]

南方医院动物实验【实验动物伦理委员会】审查意见表

The review list of the Nanfang hospital animal ethic committee to the animal protocol

申请表编号（application No）： NFYY-2017-114	计划主持人姓名(name of PI) 黄祖成 Huang Zucheng
计划名称title of the protocol：小鼠颈脊髓半侧挫伤模型的建立及其组织学特点<br>A cervical spinal cord hemi-contusion injury model in mice and its histological characterization	

(一)审 查 项 目 (item of reviews)		审查意见 Result of reviews
项次 No	实验设计及动物实验内容 design and content of the protocol	
1	该实验没有重复以前的实验内容<br>this protocol do not duplicate previous experiment.	√
2	该实验不能用非动物模型模拟动物实验<br>non-animal models cannot be used to replace the animal experiment	√
3	所选用的动物品种(系)最适合于该实验<br>the selected species is the most appropriate to this protocol	√
4	所使用的动物数量是该实验取得理想实验结果的最少数量<br>the number of animals to be used is the minimum number required to obtain valid result for this protocol	√
5	该实验设计合理，使用方法得当<br>the design is reasonable and the method is appropriate for this protocol	√
6	实验设计体现了善待动物的原则<br>The design of this protocol is accord with the principle of caring animal	√
7	动物实验人员具备动物实验资格<br>the researchers in this protocol are qualified to do this animal experiment	√
8	使用危险性物质（如生物感染性物质、放射性物质、基因重组物等）于本实验，得到本院相关部门许可，防范措施比较充分<br>using dangerous substance (such as infectious substance, radioactive substance, or genetic recombination material) is permited by related department, and the prevention is sufficient.	√
9	该实验结束后动物处理方案符合规定<br>the plan to deal with the animal in the end of the experiment is properly	√
10	该实验动物尸体、标本、废弃物的处理方案符合规定<br>the plan to dispose the carcass, specimen, waste is properly	√

(二)综合审查意见 comprehensive proposal

☑ 【通过】pass

☐ 【待修正后再审】review after amending

☐ 【不通过】no pass

理由 reasons: 所有动物实验过程符合中国及南方医院关于实验动物健康及福利的制度。

All animal care and procedures were in accordance with China and Nanfang Hospital policies for health and well-being.

动物中心主任签名 signature of laboratory animal center: [signature]	2016 年 year11 月 month 10 日 date
审查者签名 signature of the reviewers: [signatures]	2016 年 year11 月 month10 日 date
伦理委员会主任签名 signature of the director of animal ethic committee: [signature]	2016 年 year11 月 month11 日 date

(结果判别符号symbols for the judging results: √符合conform; X不符合 not conform △修正amending
