# Supplementary material for: miR-223 accelerates lipid droplets clearance in microglia following spinal cord injury by upregulating ABCA1
Source: J Transl Med. 2024 Jul 15;22:659. doi: 10.1186/s12967-024-05480-5 (PMC11247820; doi:10.1186/s12967-024-05480-5)
Supplement: Supplementary file 2 — Supplementary Material 2 [file 12967_2024_5480_MOESM2_ESM.docx]

**Supplementary Material**

**Table S1:** FDR values and log2FC for the DEMs.

| ID | FDR | log2FC | regulated |
| --- | --- | --- | --- |
| mmu-miR-106a-5p | 0.002037 | 1.877923 | up |
| mmu-miR-146a-5p | 6.24E-27 | 3.113799 | up |
| mmu-miR-152-3p | 8.71E-08 | 1.225486 | up |
| mmu-miR-155-5p | 8.63E-17 | 2.080863 | up |
| mmu-miR-199a-3p | 1.62E-07 | 1.687147 | up |
| mmu-miR-200a-3p | 0.002049 | 1.587455 | up |
| mmu-miR-200b-3p | 6.97E-05 | 1.988226 | up |
| mmu-miR-203-3p | 3.33E-10 | 1.406258 | up |
| mmu-miR-206-3p | 5.04E-10 | 1.918767 | up |
| mmu-miR-21a-5p | 1.11E-14 | 2.23577 | up |
| mmu-miR-221-3p | 1.62E-07 | 1.172681 | up |
| mmu-miR-223-3p | 0.000348 | 1.534074 | up |
| mmu-miR-23a-3p | 1.64E-10 | 1.09652 | up |
| mmu-miR-27a-3p | 6.12E-09 | 1.290429 | up |
| mmu-miR-31-5p | 1.64E-05 | 1.256153 | up |

**Table S2:** Intersecting genes and their descriptions.

| Gene symbol | Full name | Description |
| --- | --- | --- |
| ***Pgr*** | progesterone receptor | This gene encodes a member of the steroid receptor superfamily. The encoded protein mediates the physiological effects of progesterone, which plays a central role in reproductive events associated with the establishment and maintenance of pregnancy. |
| ***Syn3*** | synapsin III | Predicted to enable ATP binding activity. Involved in synaptic vesicle clustering. |
| ***Grin2b*** | glutamate receptor, ionotropic, NMDA2B (epsilon 2) | Enables NMDA glutamate receptor activity, calcium channel activity, and neurotransmitter receptor activity involved in the regulation of postsynaptic membrane potential. Involved in negative regulation of dendritic spine maintenance and positive regulation of neuron death. |
| ***Ldlr*** | low density lipoprotein receptor | Enables several functions, including amyloid-beta binding activity, low-density lipoprotein particle binding activity, and low-density lipoprotein particle receptor activity. Involved in several processes, including lipid transport, regulation of inflammatory response, and regulation of lipid metabolic process. Acts upstream of or within several processes, including cholesterol homeostasis, lipoprotein catabolic process, and low-density lipoprotein particle clearance. |
| ***Gnpda1*** | glucosamine-6-phosphate deaminase 1 | Predicted to enable glucosamine-6-phosphate deaminase activity and identical protein binding activity. Predicted to be involved in UDP-N-acetylglucosamine biosynthetic process, amino sugar catabolic process, and generation of precursor metabolites and energy. Predicted to act upstream of or within fructose 6-phosphate metabolic process, fructose biosynthetic process, and glucosamine metabolic process. |
| ***Dnm3*** | dynamin 3 | Predicted to enable several functions, including G protein-coupled glutamate receptor binding activity, GTPase activity, and nitric-oxide synthase binding activity. Predicted to be a structural constituent of postsynapse. Involved in synaptic vesicle budding from presynaptic endocytic zone membrane. |
| ***Ksr2*** | kinase suppressor of ras 2 | Enables MAP-kinase scaffold activity and mitogen-activated protein kinase kinase binding activity. Involved in positive regulation of cold-induced thermogenesis. Acts upstream of or within calcium-mediated signaling and positive regulation of MAPK cascade. |
| ***Abca1*** | ATP-binding cassette, sub-family A member 1 | The membrane-associated protein encoded by this gene is a member of the superfamily of ATP-binding cassette (ABC) transporters. ABC proteins transport various molecules across extra- and intracellular membranes. In humans, this protein functions as a cholesterol efflux pump in the cellular lipid removal pathway. Mutations in the human gene have been associated with Tangier's disease and familial HDL deficiency. |

**Figure S1:** Quality control and identification of mRNA sequencing.


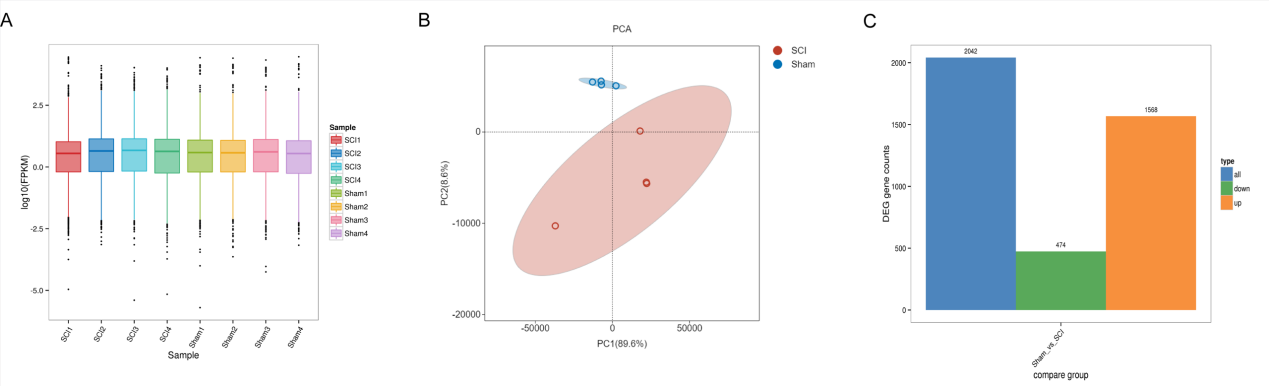


**A.** The processed data boxplot. **B.** The PCA plot. **C.** The statistical bar chart of DEGs.

**Figure S2:** GO chord of lipid-relative pathways.


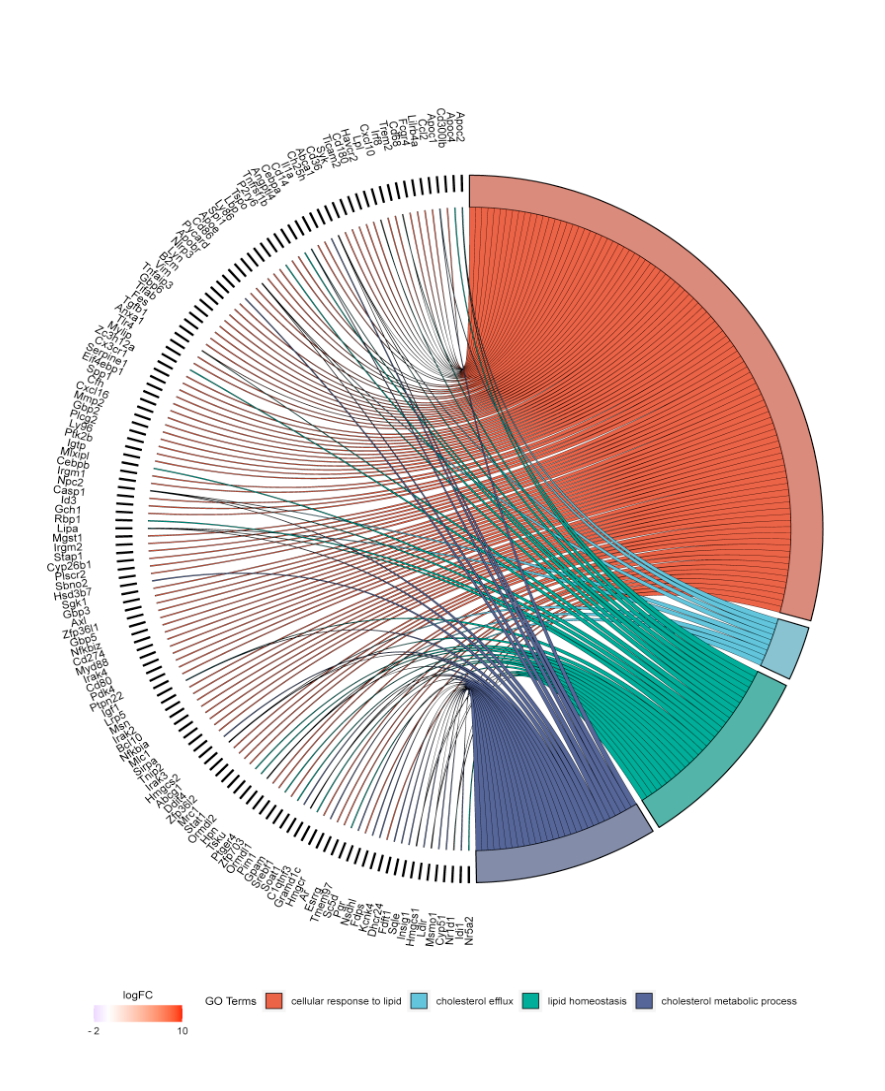


**Figure S3:** GO and KEGG pathway analyses for DEMs target genes.


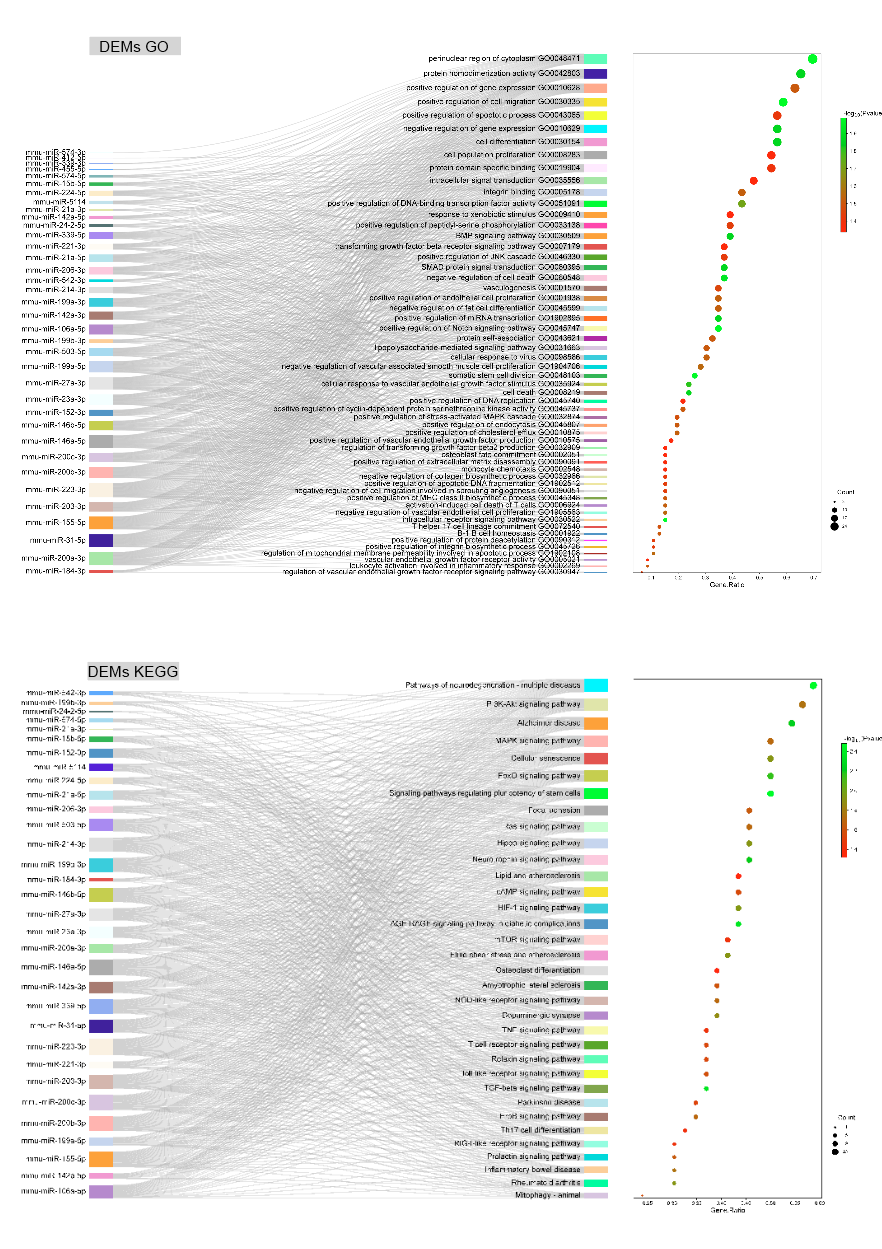


**Figure S4:** miRNA-mRNA regulatory network encompassing 85 mRNAs and 15 miRNAs.


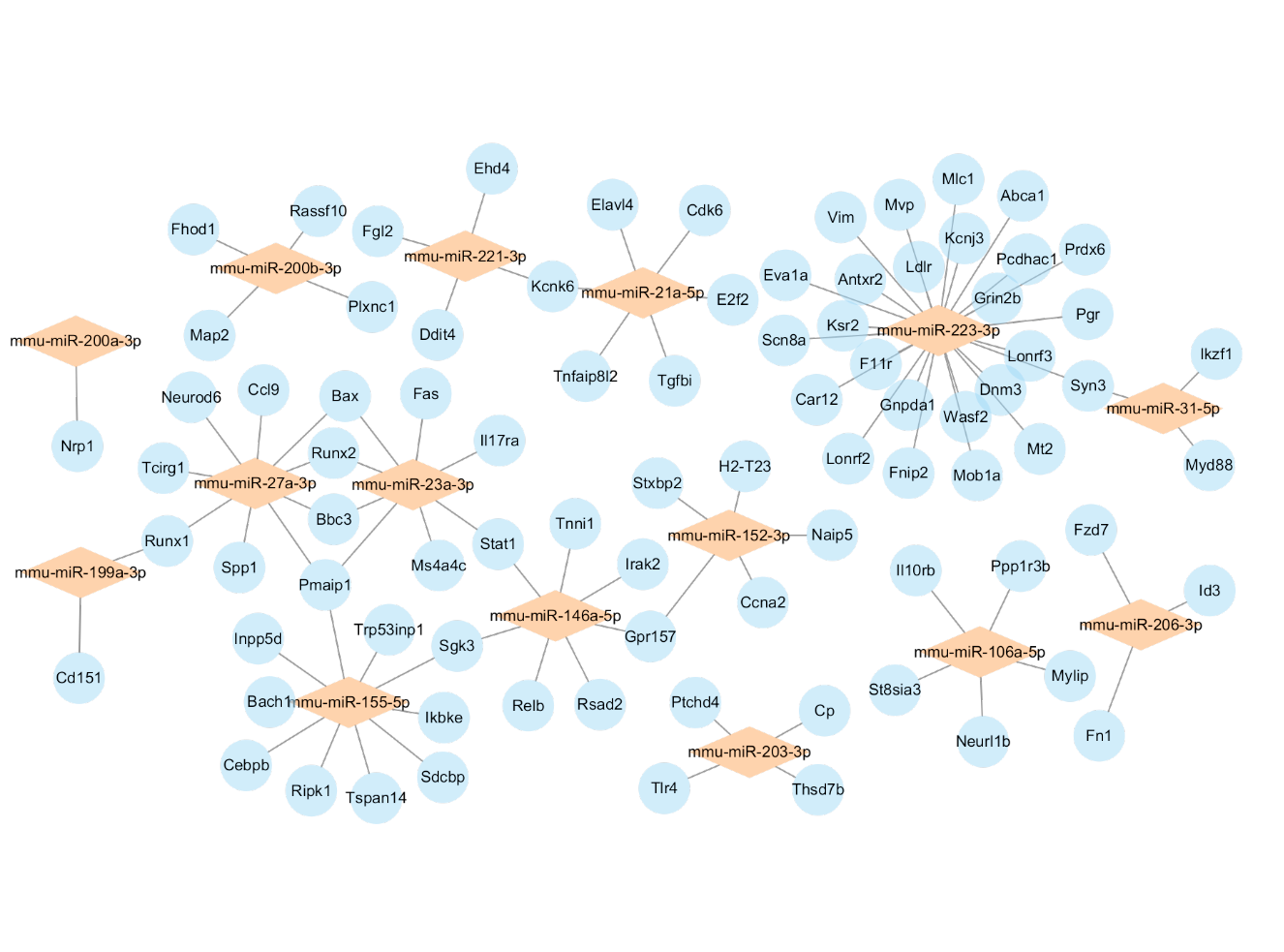


**Figure S5:** miR-223 induces upregulation of ABCA1, reduces lipid droplet accumulation, and promotes cholesterol efflux in LPS-stimulated BV2.


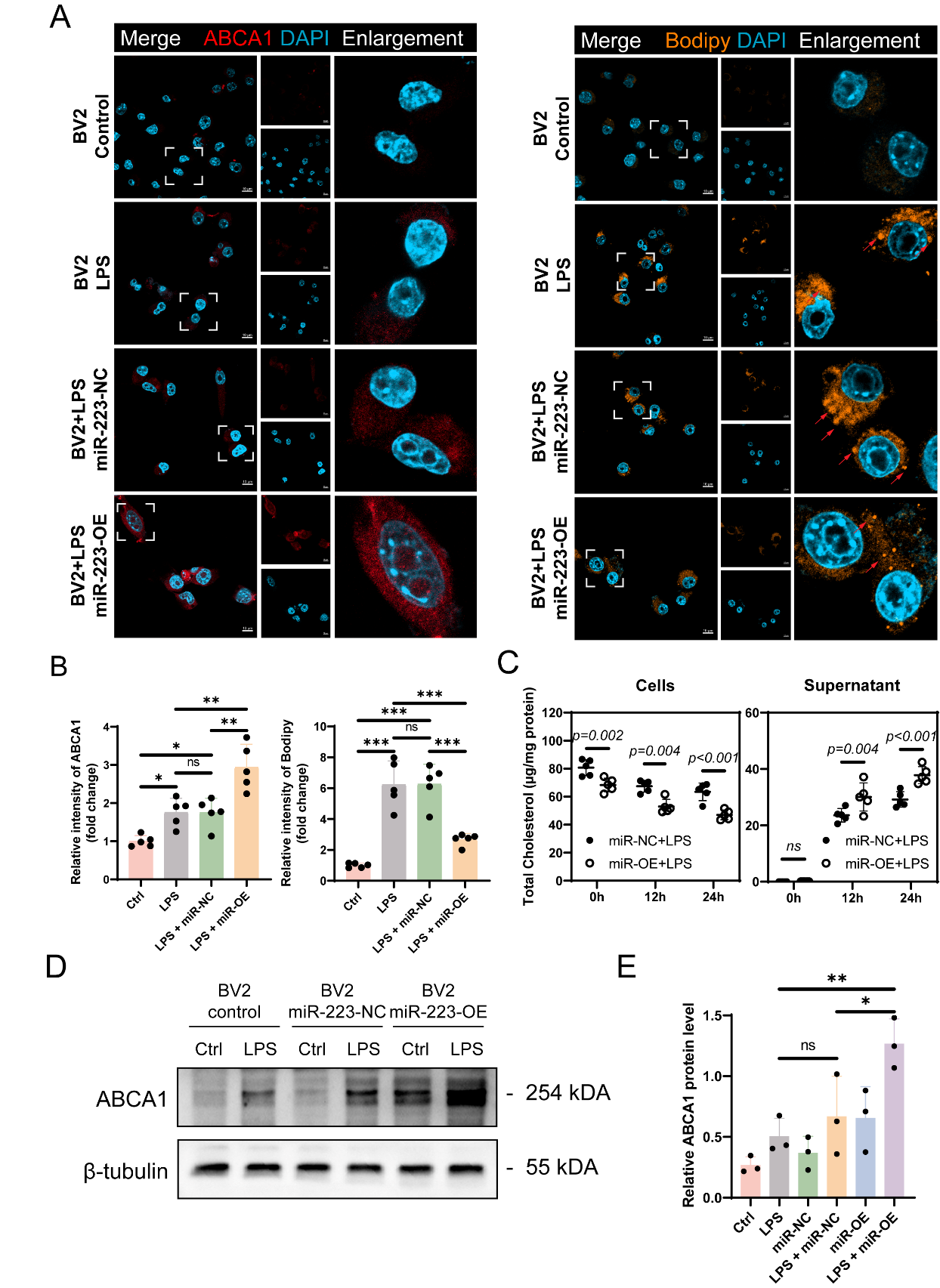


**A.** IF staining of ABCA1 (red) and LDs (BODIPY, orange) in the afore-mentioned 4 groups. **B.** Quantification analysis of MFI of ABCA1 and BODIPY (n = 5). **C.** Quantitative analysis of total cholesterol content in BV2 cells and supernatant following LPS induction (n = 5). **D/E.** Quantitative analysis of ABCA1 expression in BV2 cells using Western blot analysis normalized with the housekeeping protein β-tubulin in the six groups mentioned above (n = 3). Scale bars, 10 µm. ns: no significance, **P* < 0.05, ***P* < 0.01, ****P* < 0.001
